# Mitochondrial Mix: Combined Approach to Visualizing Oxidative Stress in Real Time

**DOI:** 10.1289/ehp.118-a304a

**Published:** 2010-07

**Authors:** Angela Spivey

**Affiliations:** **Angela Spivey** writes from North Carolina about science, medicine, and higher education. She has written for *EHP* since 2001 and is a member of the National Association of Science Writers

Oxidative stress resulting from mitochondrial dysfunction may play a role in toxicity caused by many different environmental contaminants, including aromatic hydrocarbons and metal ions, but it has been difficult to evaluate the role of mitochondria in oxidative stress using currently available methods. A new study combining new and established imaging techniques to document mitochondrial dysfunction now indicates this integrated approach to real-time imaging in live cells can be useful for studying the role of oxidative stress in toxicologic responses **[*EHP*** **118(7):902–908; Cheng et al.]**.

Real-time imaging in live cells to detect products of oxidative stress known as reactive oxygen species (ROS) offers superior temporal and spatial resolution compared with traditional methods such as detecting oxidized lipids, proteins, or DNA. But the accuracy and utility of the fluorescent indicator H2DCF-DA, a reagent commonly used for directly detecting ROS in living cells, is limited.

The authors conducted a set of experiments in which they exposed cultured human skin carcinoma cells to zinc (Zn^2+^), a ubiquitous contaminant known to induce oxidative stress. Three different fluorescent imaging techniques were used to study effects of Zn^2+^ on mitochondria. The first used the fluorophore PG1 to measure production of the ROS hydrogen peroxide. The scientists found that hydrogen peroxide increased within the cells upon Zn^2+^ exposure and that its production was inhibited with the addition of the mitochondrial inhibitor CCCP, implicating mitochondria as the source of the Zn^2+^-induced hydrogen peroxide.

A second experiment used the fluorescent indicator JC-1 to measure changes in mitochondrial membrane potential (the difference in electrical potential between the inside and outside of the mitochondrial membrane) following exposure to Zn^2+^. When Zn^2+^ was administered, loss of JC-1 fluorescence emission indicated a loss of membrane potential consistent with impaired mitochondrial function.

A third experiment used the genetically encoded fluorescent sensor MTroGFP1 to measure the redox potential of mitochondria after Zn^2+^ exposure. MTroGFP1 associates with mitochondria in transfected cells, causing the mitochondria to fluoresce. However, the investigators observed a change in the fluorescent signals following exposure to Zn^2+^ consistent with a loss of redox potential. In a fourth experiment on mitochondria isolated from live mouse hearts, the authors demonstrated that administering Zn^2+^ resulted in rapid mitochondrial swelling, another indication of a loss of mitochondrial function.

The study shows the value of combining multiple imaging techniques to constitute an integrated approach that permits real-time monitoring of the mechanisms behind oxidative stress within living cells. The results also add to the evidence that oxidative stress induced by Zn^2+^ originates in mitochondria and sheds light on some of the mechanisms that may be involved. Further study is under way to determine the exact sequence of cellular events by which toxicants induce generation of ROS and mitochondrial dysfunction.

